# A Robust Vision-Based Method for Displacement Measurement under Adverse Environmental Factors Using Spatio-Temporal Context Learning and Taylor Approximation

**DOI:** 10.3390/s19143197

**Published:** 2019-07-20

**Authors:** Chuan-Zhi Dong, Ozan Celik, F. Necati Catbas, Eugene OBrien, Su Taylor

**Affiliations:** 1 Department of Civil, Environmental, and Construction Engineering, University of Central Florida, 12800 Pegasus Drive, Suite 211, Orlando, FL 32816, USA; 2 The School of Civil Engineering, University College Dublin, Belfield D04V1W8, Ireland; 3 The School of Natural and Built Environment, Queens University Belfast, Belfast BT95AG, UK

**Keywords:** structural health monitoring, displacement measurement, non-contact, computer vision, environmental factors, spatio-temporal context, Taylor approximation

## Abstract

Currently, the majority of studies on vision-based measurement have been conducted under ideal environments so that an adequate measurement performance and accuracy is ensured. However, vision-based systems may face some adverse influencing factors such as illumination change and fog interference, which can affect measurement accuracy. This paper developed a robust vision-based displacement measurement method which can handle the two common and important adverse factors given above and achieve sensitivity at the subpixel level. The proposed method leverages the advantage of high-resolution imaging incorporating spatial and temporal contextual aspects. To validate the feasibility, stability, and robustness of the proposed method, a series of experiments was conducted on a two-span three-lane bridge in the laboratory. The illumination changes and fog interference were simulated experimentally in the laboratory. The results of the proposed method were compared to conventional displacement sensor data and current vision-based method results. It was demonstrated that the proposed method gave better measurement results than the current ones under illumination change and fog interference.

## 1. Introduction

### 1.1. Background

Computer vision-based displacement measurement using cameras has attracted increasing attention in the community of structural health monitoring (SHM) because of its characteristics as a non-contact, long-distance, multi-point, high-precision, time-saving, and cost-effective sensing technique [1,2,3,4,5,6,7,8,9,10,11,12,13,14,15,16,17]. Structural displacement is a critical indicator for evaluating performance and identifying and determining the effects of damage/change under external loads [18,19]. For instance, during the regular operation of a structure, displacement can be monitored to ensure that it stays within a specified tolerance and safety range [20]. Once the displacement time histories from the monitored structures are extracted using vision-based methods, traditional structural health monitoring and behavior analysis can easily be conducted [21,22]. Vision-based displacement measurement methods are also applied for bridge load testing to evaluate the bridge load carrying capacity [23] and have even been used for contactless bridge weigh-in-motion [24]. Combining the multi-point displacement response with structural input data extracted from vehicle tracking, structural identification can be carried out using traditional structural indicators such as the unit influence line (UIL) and unit influence surface (UIS) [25,26,27]. Without the need for the deployment of conventional sensor networks, operational modal analysis can be performed using vision-based displacement measurement methods, which may provide multi-point synchronization and, therefore, a much denser spatial resolution than is practical with conventional sensors [12,28,29,30,31,32,33]. Full field motion estimation and instantaneous mode shapes can even be obtained with high spatial and temporal resolution [21,34,35,36]. Reference [33] introduced an application based on motion magnification for modal identification of an on-the-field, full-scale, large historic masonry bridge by using videos taken from a common smartphone device. Modal properties and other indices derived from vision-based displacement time histories can be turned into sensitive indicators for structural damage detection and model updating [37,38,39]. There are also numerous studies related to the estimation of stay cable forces that use vision-based displacement measurement [40,41]. In addition to structural response monitoring, the external loading information can be predicted. Celik et al. [7] estimated the load time histories of individuals and crowds with the displacement time histories obtained using computer vision-based measurements. These successful research applications make computer vision-based displacement methods a very promising complementary tool to conventional structural health monitoring practices, particularly for bridges.

### 1.2. Motivations and Objectives

The majority of applications and experiments in the literature are conducted in an ideal measurement environment so that an adequate measurement performance and accuracy is ensured. In addition, when these experiments are performed for the purposes of new method verification or comparison, the measurement time span is generally short and the adverse factors which can influence the measurement accuracy and stability are mostly avoided. For a general proof of concept, it makes sense to conduct such studies. However, when vision-based systems are intended for long-term deployment, either as a standalone or to complement a conventional SHM system, some unfavorable contingencies may affect the measurement quality. Even in the short term, the accuracy and stability of a vision-based system can be affected adversely. In a review of the current literature, Feng and Feng [16] summarized the possible measurement error sources in vision-based methods, including: (1) camera motion; (2) coordinate conversion; (3) hardware limitations; and (4) environmental sources. Brownjohn et al. [5] investigated the challenges in field application of a commercial vision-based system resulting from camera instability, the nature of the target (artificial or structural feature), and illumination. Ye et al. [42] reviewed the state-of-the-art systematic errors, assessment, and reduction, including: (1) target size and texture; (2) camera alignment; (3) motion blur; and (4) the ratio between target size and full view. Xu and Brownjohn [4] reviewed subpixel techniques used in vision-based displacement measurement methods. Ma et al. [43] studied the measurement error in the digital image correlation method caused by the self-heating of digital cameras. Ye et al. [44] conducted a series of shaking table experiments in the laboratory to examine the influence of environmental factors which affect the accuracy and stability of vision-based systems. The targets used in the experiments were QR (quick response) codes and the textures of the QR codes showed rich sparkle patterns. It is suggested that measurement results are adversely affected by illumination and vapor. Subsequently, Dong and Ye [45,46] investigated the possibility of improving the accuracy and the stability of vision-based systems by mitigating the adverse effects of vapor. They used light emitting diodes (LEDs) and infrared emitting diodes as the measurement target and the experimental results showed that these emitting diodes can mitigate the adverse effects of vapor. However, installing these kinds of targets on the structure can be difficult, perhaps requiring wiring and a main power supply, which may not be feasible for a bridge.

These problems may decrease the accuracy of the measurement results, and affect the evaluation of structural performance and health conditions when using vision-based monitoring systems over long-term time spans. In the literature, there are lots of studies on the analysis of sources of error, but only a few [42,44] seek to improve system performance under adverse influencing factors. Therefore, it is essential to develop a robust vision-based displacement measurement method for long-term structural monitoring, which can handle some of these adverse factors.

While one study cannot address all issues related to computer vision-based monitoring, this paper focused on the mitigation of environmental factors such as illumination change and fog interference, and improvement of the measurement sensitivity at the subpixel level. A robust vision-based displacement measurement method was developed, leveraging the advantages of high-resolution imaging and computer-vision techniques to mitigate the interferences induced by illumination change and fog, and was adapted for long-term bridge monitoring. The proposed method utilizes the spatio-temporal context (STC) learning algorithm to track measurement objects in image sequences and obtain the locations. The STC algorithm [47] builds the spatio-temporal relationships between the measurement target and its local context based on a Bayesian framework, which models the statistical correlation between the low-level features (i.e., image intensity and position) from the measurement target and its surrounding regions. The tracking problem is solved by computing a confidence map and obtaining the best target location by maximizing an object location likelihood function. Combining this with the Taylor approximation [48], the accuracy of the proposed method achieves a subpixel level without sacrificing processing speed. The objectives of this study were: (1) developing a new vision-based displacement method using spatio-temporal context learning; (2) achieving a subpixel level estimation based on a Taylor approximation for the new vision-based method; and (3) verifying the feasibility, stability, and robustness of the proposed method via comparison with the current vision-based methods and conventional displacement sensor (Linear Potentiometer, LP) by conducting a series of experiments under two adverse environmental factors (illumination change and fog) on a two-span three-lane model bridge in the laboratory.

## 2. Methodology

### 2.1. General Procedure of the Vision-Based Displacement Measurement Methods

**Figure 1:**
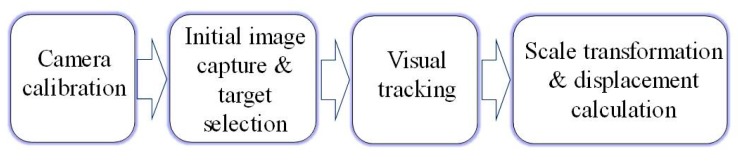
General procedure of the vision-based displacement measurement methods.

The key aspect of vision-based displacement measurement methods is to convert the measurement of the target motion in the image into actual motion with physical units such as millimeters. In the literature, researchers have proposed different procedures for vision-based displacement measurement. In this study, the authors summarized the state-of-the-art methods and present a general procedure as illustrated in Figure 1. There are four steps in this procedure, namely: (1) camera calibration; (2) initial image capture and target selection; (3) visual tracking and (4) scale transformation and displacement calculation.

In the first step, with the camera calibration, the relationship between the image entity and physical world object is built. A camera is set up with the measurement targets included in the field of view. This is also the step where camera calibration is conducted. If the camera is wide angle, it can cause image distortion [49]. Yoon et al. [6] and Brownjohn et al. [5] implement a flexible technique which was proposed by using the camera to observe a planar calibration object in a few different views to easily calibrate a camera and resolve the distortion problems. The authors also recommend implementing this method to resolve the distortion problems. In this study, the authors selected a distortion-free camera so that distortion problems were not an issue. With Zhang’s method [49], it is also possible to convert the image entities into physical world objects, since during the camera calibration, the planar homography matrix between the image and the physical world is obtained. In this paper, instead of a planar homography matrix, for convenience, the authors used the scale factor to convert the target motion in pixels to physical units of millimeters. When the optical axis is perpendicular to the object motion plane, the scale factor, *s*, is:

(1) $$s=\frac{D}{d}$$

where *D* is the physical length of the object on the motion plane and *d* is the length in pixels of its corresponding image part. When there is an angle between the target motion plane and optical axis, *θ*, the scale factor has to be modified by:

(2) $$s=\frac{D}{d\cos\theta }$$

More details about the scale factor calculation can be found in the literature [7,16,20,40,42,44,45,46,50,51].

In the second step, the region to be tracked, which includes the measurement targets in the field of view of the initial image, is selected. According to the visual tracking methods used in the third step, image preprocessing is utilized to extract useful features from the tracking region. Researchers who use digital image correlation (DIC) either in the frequency domain [14] or the spatial domain [32] select the target regions as the patterns and the low-level features of images play the role of visual tracking features. Other researchers extract key points and descriptors, such as Shi-Tomasi corners, SURF (speeded-up robust features), SIFT (scale-invariant feature transform), FREAK (fast retina keypoint), etc., as the tracking features and the corresponding visual tracking method includes Lucas–Kanade optical flow estimation and key point matching based on nearest neighborhood approximation [3,6,17]. The third step is to track the selected targets in subsequent images captured by the camera continuously and locate the targets’ new positions. In this paper, the authors implemented the spatio-temporal context (STC) learning method to conduct the visual tracking. The horizontal and vertical displacements in pixels—*x_t_* − *x*_0_ and *y_t_* − *y*_0_, respectively—are found by subtracting the coordinates of the initial target position (*x*_0_, *y*_0_) from the current target position (*x*_t_, *y_t_*). When pattern matching methods such as DIC are used in this step, the displacements in pixels are always integer values. One way to increase the sensitivity and to improve the measurement accuracy is by applying subpixel techniques. For instance, Feng et al. [14] implement upsampled cross correlation in the local region to obtain the displacement at the subpixel level. In this paper, the authors utilized the Taylor approximation method to achieve the subpixel level without upsampling the images and without sacrificing the image processing speed. Finally, with the scale factor, *s*, and the displacement in pixels, the actual displacement at time *t* of the physical unit is obtained: (*x_t_* − *x*_0_)*s*, horizontally, and (*y_t_* − *y*_0_)*s*, vertically. The visual tracking method and subpixel estimation used in this paper are introduced in detail in the next sections.

### 2.2. Visual Tracking Using Spatio-Temporal Context (STC) Learning

**Figure 2:**
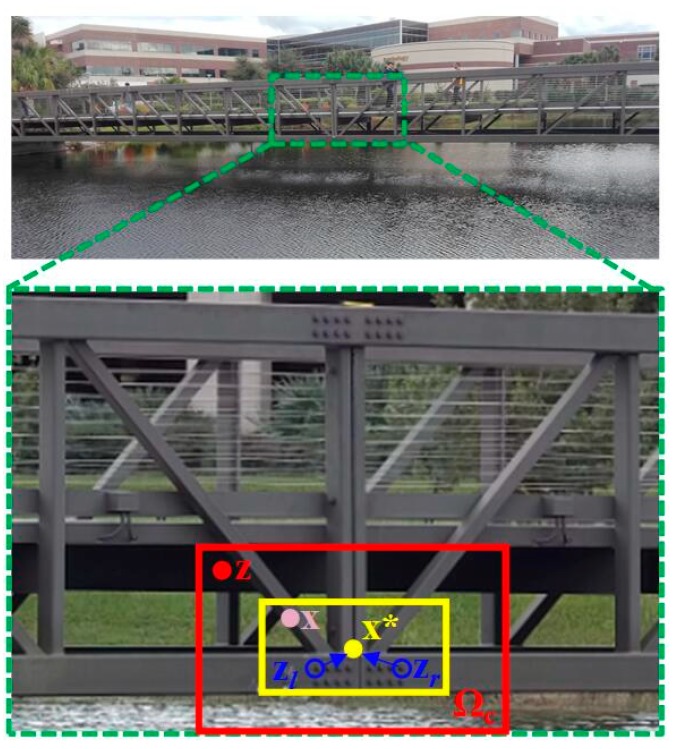
Graphical model of spatial relationship between the target and its surrounding context.

The spatio-temporal relationship among the local scenes containing the target in consecutive frames can be used to model the statistical correlation between the low-level features, such as image intensity and position, extracted from the target and its local context [47]. As illustrated in the footbridge example of Figure 2, the yellow (smaller) box is the target to be tracked and the red (larger) box is the local context.

The visual tracking task can be obtained by maximizing an object location likelihood function c(**x**) as [47]:

(3) $$\begin{array}{ll} c\left(x\right) & =P\left(x|o\right)=\sum_{c\left(z\right)\in X^{c}}P\left(x,c\left(z\right)|o\right)\\ & =\sum_{c\left(z\right)\in X^{c}}P\left(x|c\left(z\right),o\right)P\left(c\left(z\right)|o\right) \end{array}$$

where **x** is the target location which can be represented with the coordinates defined above, (*x*, *y*) and *o* denotes the target present in the scene. The context feature set, *X^c^*, is defined as:

(4) $$X^{c}=\left\{c\left(z\right)=\left(I\left(z\right),z\right)|z\in \Omega_{c}\left(x^{*}\right)\right\}$$

where *I*(**z**) denotes the image intensity at location **z** and Ω_c_(x^*^) is the neighborhood of location x^*^. The conditional probability *P*(**x**|**c**(**z**), *o*) in Equation (3) models the spatial relationship between the object location and its context information. It can help to resolve ambiguities when the image measurements allow different interpretations, which are introduced in the following parts. *P*(**c**(**z**)|*o*) is a context prior probability which models the appearance in the local context.

The conditional probability *P*(**x**|**c**(**z**), *o*) in Equation (3) is defined as:

(5) $$P\left(x|c\left(z\right),o\right)=h^{sc}\left(x-z\right)$$

where *h^sc^*(**x** − **z**) is the spatial context model function, which is only of the direction and the relative distance between the target location **x** and its local context location **z**, which means this function contains the spatial relationship between the target and its spatial context. Equation (5) defines the spatial context model. It is worth noting that Equation (5) is not a radially symmetric function which means that *h^sc^*(**x** − **z**) is not equal to *h^sc^*(|**x** − **z**|). It considers different spatial relationships between the target and its local context, which facilitates the solving of the ambiguities when similar objects appear in close proximity. As shown in Figure 2, when a visual tracking method tries to track a bolt based only on the appearance denoted by **z***_l_*, it might be distracted to the right one denoted by **z***_r_*, because both bolts and the local surroundings have a similar appearance. This would cause ambiguities and consequently decrease the tracking accuracy, especially when the target moves fast, and the search region is not small. With the non-radially symmetric characteristics of *h^sc^*(**x** − **z**), the ambiguities can be resolved.

In Equation (3), *P*(**c**(**z**)|*o*) can be calculated according to the target location that has been initialized manually in the first frame. It is modeled by:

(6) $$P\left(c\left(z\right)|o\right)=I\left(z\right)w_{\sigma }\left(z-x^{*}\right)$$

where *w_σ_*(·) is a weighted function defined by:

(7) $$w_{\sigma }\left(z\right)=ae^{-\frac{{\left|z\right|}^{2}}{\sigma^{2}}}$$

In Equation (7), *a* is the normalization constant which restricts *P*(**c**(**z**)|*o*) to be in the range from 0 to 1 and *σ* is a scale parameter. Equation (6) ensures that, the closer the context location **z** is to the current tracking target location x^*^, the more important it is to predict the location and a greater weight is set.

The confidence map of an object location is modeled as:

(8) $$c\left(x\right)=P\left(x|o\right)=be^{-{\left|\frac{x-x^{*}}{\alpha }\right|}^{\beta }}$$

where *b* is a normalization constant, *α* is a scale parameter, and *β* is a shape parameter. According to the literature [47], robust results can be obtained when *β* = 1. Based on the context prior model in Equation (6) and the confidence map function in Equation (8), the objective is to learn the spatial context model, i.e., Equation (3). Combining Equations (3), (5), (6) and (8), gives:

(9) $$\begin{array}{ll} c\left(x\right) & =P\left(x|o\right)=be^{-{\left|\frac{x-x^{*}}{\alpha }\right|}^{\beta }}\\ & =\sum_{z\in \Omega_{c}\left(x^{*}\right)}h^{sc}\left(x-z\right)I\left(z\right)w_{\sigma }\left(z-x^{*}\right)\\ & =h^{sc}\left(x\right)⊗I\left(x\right)w_{\sigma }\left(x-x^{*}\right) \end{array}$$

where ⊗ denotes the convolution operator. The fast Fourier transform (FFT), Equation (9) transforms the function to the frequency domain:

(10) $$ℱ\left(be^{-{\left|\frac{x-x^{*}}{\alpha }\right|}^{\beta }}\right)=ℱ\left(h^{sc}\left(x\right)\right)⊙ℱ\left(I\left(x\right)w_{\sigma }\left(x-x^{*}\right)\right)$$

where *ℱ* denotes the FFT function and ⊙ is the element-wise product. Then, the spatial context model is:

(11) $$h^{sc}\left(x\right)={ℱ}^{-1}\left(\frac{ℱ\left(be^{-{\left|\frac{x-x^{*}}{\alpha }\right|}^{\beta }}\right)}{ℱ\left(I\left(x\right)w_{\sigma }\left(x-x^{*}\right)\right)}\right)$$

where *ℱ*^−1^ denotes the inverse FFT function. Then in the whole image sequence, the spatio-temporal context model of the (*t* + 1)th frame, $H_{t+1}^{stc}$, can be updated using the spatio-temporal context model, $H_{t}^{stc}$, and the spatial context model, $h_{t}^{sc}$, of the *t*th frame. It is formulated as:

(12) $$H_{t+1}^{stc}=\left(1-\rho \right)H_{t}^{stc}+\rho h_{t}^{sc}$$

where *ρ* is a learning parameter and *t* denotes the *t*th frame. It should be noted that in the first frame, i.e., when *t* is equal to 1, the spatio-temporal context model $H_{t}^{stc}$ is equal to the spatial context model, $h_{t}^{sc}$.

Finally, the target location $x_{t+1}^{*}$ in the (*t* + 1)th frame is determined by maximizing the new confidence map:

(13) $$x_{t+1}^{*}=\underset{x\in \Omega_{c}\left(x_{t}^{*}\right)}{\arg\max}c_{t+1}\left(x\right)$$

Deduced from Equation (10), the new confidence map *c_t_*_+1_(**x**) is represented as:

(14) $$c_{t+1}\left(x\right)={ℱ}^{-1}\left(ℱ\left(H_{t+1}^{stc}\left(x\right)\right)⊙ℱ\left(I_{t+1}\left(x\right)w_{\sigma_{t}}\left(x-x^{*}\right)\right)\right)$$

As the scale of the target may tend to change over time, the scale parameter *σ* in the weight function *w_σ_* in Equation (7) is updated by:

(15) $$\{\begin{array}{c} s_{t}^{\prime }=\sqrt{\frac{c_{t}\left(x_{t}^{*}\right)}{c_{t-1}\left(x_{t-1}^{*}\right)}}\\ {\bar{s}}_{t}=\frac{1}{n}\sum_{i=1}^{n}s_{t-i}^{\prime }\\ s_{t+1}=\left(1-\lambda \right)s_{t}+\lambda {\bar{s}}_{t}\\ \sigma_{t+1}=s_{t}\sigma_{t} \end{array}$$

where $s_{t}^{\prime }$ is the estimated scale among two consecutive frames. The estimated target scale *s_t_*_+1_ is obtained through filtering, in which ${\bar{s}}_{t}$ is the average of the estimated scale from *n* consecutive frames to avoid oversensitive adaptation and to reduce noise, and *λ* > 0 is a fixed parameter. More details about the derivation can be found in the literature [47]. In general, the scale updating should be considered, but in this study, only in-plane motion is considered for two-dimensional displacement measurement, so that scale updating is neglected. If this method is used to do three-dimensional displacement measurement, which means there is out-plane motion, scale updating has to be considered.

To obtain robust tracking results, the reference gives rules of thumb regarding the selection of the parameters used in STC tracking: *α* = 2.25, *β* = 1, *ρ* = 0.075, *s*_1_ = 1, *λ* = 0.25, and *n* = 5. Additionally, for Equation (12), with the deductions,

$$\begin{array}{ccc} H_{t+1}^{stc} & = & \left(1-\rho \right)H_{t}^{stc}+\rho h_{t}^{sc};\\ \int H_{t+1}^{stc}e^{-jwt}dt & = & \int \left(1-\rho \right)H_{t}^{stc}e^{-jwt}dt+\int \rho h_{t}^{sc}e^{-jwt}dt; \end{array},\;\begin{array}{ll} \mathrm{LHS} & =\int H_{t+1}^{stc}e^{-jwt}dt\overset{t\to t-1}{=}\int H_{t}^{stc}e^{-jw\left(t-1\right)}d\left(t-1\right)\\ & =e^{jw}\int H_{t}^{stc}e^{-jwt}dt=e^{jw}H_{w}^{stc};\\ \mathrm{RHS} & =\int \left(1-\rho \right)H_{t}^{stc}e^{-jwt}dt+\int \rho h_{t}^{sc}e^{-jwt}dt\\ & =\left(1-\rho \right)\int H_{t}^{stc}e^{-jwt}dt+\rho \int h_{t}^{sc}e^{-jwt}dt\\ & =\left(1-\rho \right)H_{w}^{stc}+\rho h_{w}^{sc}; \end{array}\;\begin{array}{l} \mathrm{LHS}=\mathrm{RHS};\\ H_{w}^{stc}=\frac{\rho }{e^{jw}-\left(1-\rho \right)}h_{w}^{sc} \end{array}$$

a temporal filtering procedure can be easily obtained in the frequency domain, which is:

(16) $$H_{w}^{stc}=\frac{\rho }{e^{jw}-\left(1-\rho \right)}h_{w}^{sc}$$

where:

(17) $$H_{w}^{stc}=\int H_{t}^{stc}e^{-jwt}dt$$

is the temporal Fourier transform of $H_{t}^{stc}$ and similar to $h_{w}^{sc}$. The temporal filter can be represented by:

(18) $$F_{w}=\frac{\rho }{e^{jw}-\left(1-\rho \right)}$$

which is a low-pass filter [52]. With this low-pass filter, the spatio-temporal context model is able to filter out image noise caused by appearance variations and this leads to more stable measurement results. The properties of the spatio-temporal model contribute to the resolution of the adverse effects induced by environmental factors such as illumination change and fog.

### 2.3. Subpixel Level Estimation Using Taylor Approximation

With Equation (13), the targets can easily be tracked in the image sequence, but the change of locations can only be obtained with integer pixel values. To achieve subpixel level motion, the Taylor approximation method is applied to solve the optical flow estimation. Assuming there are two consecutive images, *f*(*x*, *y*) and *g*(*x*, *y*), with a shift (Δ*x*, Δ*y*), the following estimation applies:

(19) $$\begin{array}{l} \;g\left(x,y\right)=f\left(x+\Delta x,y+\Delta y\right)\\ \approx f\left(x,y\right)+\Delta x\frac{\partial }{\partial x}f\left(x,y\right)+\Delta y\frac{\partial }{\partial y}f\left(x,y\right) \end{array}$$

which is the first order Taylor series approximation. The shift in the image can be calculated by minimizing the sum of squared errors (SSEs):

(20) argminΦ(Δx,Δy)

where:

(21) $$\;\Phi \left(x,y\right)=\sum_{x,y}{\left[g\left(x,y\right)-f\left(x,y\right)-\Delta x\frac{\partial }{\partial x}f\left(x,y\right)-\Delta y\frac{\partial }{\partial y}f\left(x,y\right)\right]}^{2}\;$$

Using the ordinary least squares (OLS) method to solve Equation (20), the optimal Δ*x* and Δ*y* can be determined by setting the partial derivatives of Equation (21) to zero, i.e.,

(22) $$\{\begin{array}{c} \frac{\partial \Phi }{\partial \Delta x}=0\\ \frac{\partial \Phi }{\partial \Delta y}=0 \end{array}\;$$

Combining Equations (21) and (22) gives the system of linear equations:

(23) $$\left[\begin{array}{cc} \sum_{x,y}{\left(\frac{\partial f}{\partial x}\right)}^{2} & \sum_{x,y}\frac{\partial f}{\partial x}\frac{\partial f}{\partial y}\\ \sum_{x,y}\frac{\partial f}{\partial x}\frac{\partial f}{\partial y} & \sum_{x,y}{\left(\frac{\partial f}{\partial y}\right)}^{2} \end{array}\right]\left\{\begin{array}{c} \Delta x\\ \Delta y \end{array}\right\}=\left\{\begin{array}{c} \sum_{x,y}\left(g-f\right)\frac{\partial f}{\partial x}\\ \sum_{x,y}\left(g-f\right)\frac{\partial f}{\partial y} \end{array}\right\}$$

The optimal shift, (Δ*x*, Δ*y*), is obtained by solving Equation (23). It should be noted that to make the Taylor approximation valid, the assumptions |Δ*x*| << 1 and |Δ*y*| << 1 have to be satisfied. When small motion is estimated, i.e., motion less than one pixel, the assumption holds. The procedure simplified from optical flow estimation is called Taylor approximation here and it will be utilized to solve the subpixel level motion estimation [43].

**Figure 3:**
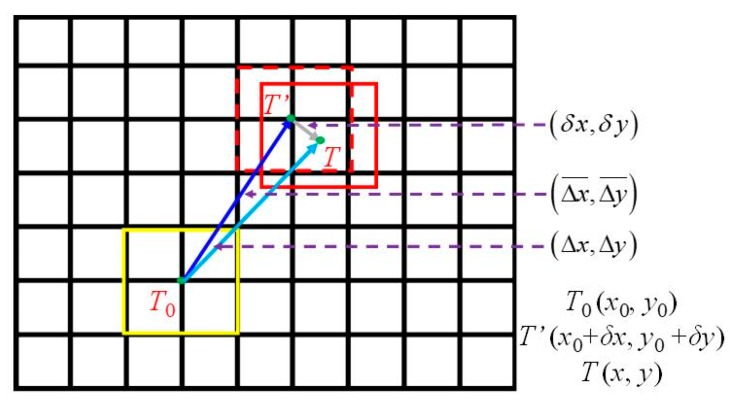
Sketches of motion estimation using spatio-temporal context (STC) tracking and Taylor approximation.

**Figure 4:**
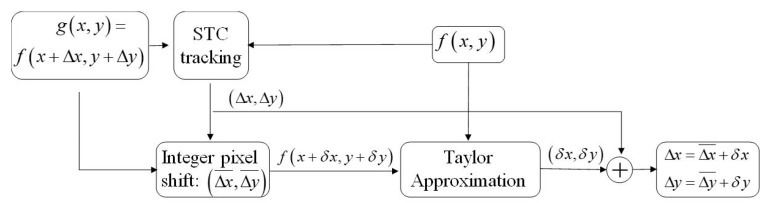
Flowchart of STC-based subpixel tracking using Taylor approximation.

Figure 3 and Figure 4 illustrate the proposed motion estimation at the subpixel level. At first, the spatio-temporal context (STC) tracking method is employed to determine the integer pixel displacements, ($\bar{\Delta x}$, $\bar{\Delta y}$).

In Figure 3, the yellow, solid line box represents the original target location in the initial frame and the red, dashed line box represents the target recognized in the current frame using STC tracking, which has an accuracy at the subpixel level. Here the centers of the targets are used to represent their locations, i.e., *T*_0_ and *T’*. Assuming the real target in the current frame is the red, solid line box at location *T*, the true displacements are (Δ*x*, Δ*y*). Then, the displacements ($\bar{\Delta x}$, $\bar{\Delta y}$) are the integer estimations of the true displacements, (Δ*x*, Δ*y*). The differences between ($\bar{\Delta x}$, $\bar{\Delta y}$) and (Δ*x*, Δ*y*) are (*δx*, *δy*), from *T’* to *T*, where |δx| < 1 and |*δy*| < 1, and the assumption of using the Taylor approximation is satisfied with the conditions of |*δx*| < 1 and |*δy*| < 1. Secondly, the Taylor approximation is employed to estimate the displacement between the target tracked by STC (red, dashed line box) and the real target (in red, solid line box), i.e., (*δx*, *δy*). Finally, the total displacements are:

(24) $$\{\begin{array}{c} \Delta x=\bar{\Delta x}+\delta x\\ \Delta y=\bar{\Delta y}+\delta y \end{array}$$

According to the literature [48], the Taylor approximation gives an error bound of less than 0.0125 pixels, without any image upsampling and the error is much smaller than that of the normal template matching methods using image upsampling. The feasibility and performance of the proposed method for structural displacement will be verified through laboratory experiments in the next sections.

## 3. Laboratory Verification

### 3.1. Experimental Setup

**Figure 5:**
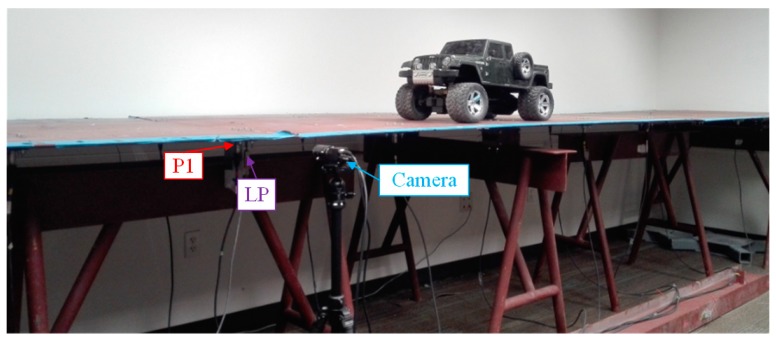
Experimental setup.

Figure 5 shows the two-span bridge model constructed at the University of Central Florida’s (UCF) Civil Infrastructure Technologies for Resilience and Safety (CITRS) Experimental Design and Monitoring (EDM) laboratory. The bridge was a scaled down model of a mid-sized real-life structure and a toy truck with variable weights was used to model moving loads. The bridge consisted of two 300 cm main continuous spans, which were rebuilt from UCF’s original four-span bridge [17]. The bridge deck, which included a 3.18 mm steel sheet, was 120 cm wide and 600 cm long. The steel deck was supported by two 25 mm × 25 mm × 3.2 mm steel girders separated 0.61 m from each other. To provide a connection between girders and the deck, a set of four M6 bolts and 3.18 mm thick plates were utilized. The total length of the toy truck was 68.6 cm and the distance among the two axles was 45.72 cm. The width and the height of the toy truck were 35.6 cm and 33.1 cm, respectively. The total weight of the truck was 13.48 kg with a front axle weight of 1.9 kg and a rear axle weight of 11.58 kg. The average moving speed of the toy truck was manually controlled to achieve 0.4 m/s, while the actual speed in the experiment for three cases were 0.36 m/s, 0.38 m/s, and 0.33 m/s, respectively.

**Figure 6:**
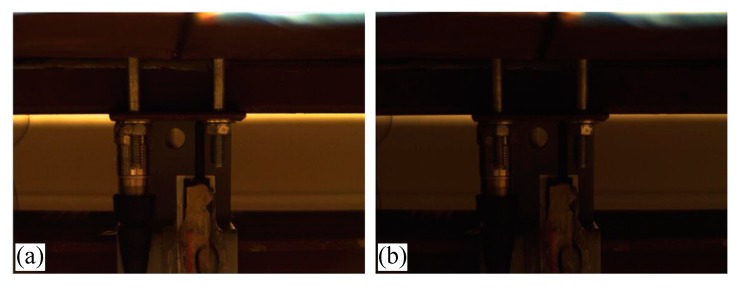
Illumination change. (**a**) 34 lux, (**b**) 18 lux.

**Figure 7:**
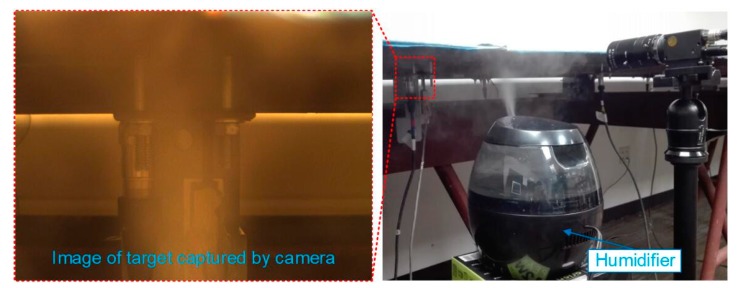
Fog simulation.

An industrial camera was set-up in front of the bridge to record images at a measuring point (P1) during the moving load trials. A linear potentiometer (LP) was mounted under the deck to measure the displacement of the P1 and was assumed as the ground truth. The model number was BEI Duncan 9615. Detailed information about the sensor can be found in Reference [53]. During the experiments, the truck moved from one side of the bridge to the other while the LP and the camera recorded the motion of the P1 (midspan of the left span) synchronously. The resolution of the camera was 1280 × 960, with a maximum frame rate of 60 FPS (frames per second). Here, the frame rate was set to 30 FPS. The focal length of the lens was within a zoom range of 6~60 mm. The sampling rate of the data acquisition system for the LP was 300 Hz, which was then downsampled to 30 Hz during post processing. Three experimental cases were specified to achieve the objectives of this paper:

Case 1: The truck moves on the bridge in ideal conditions and no adverse factors are imposed in the measuring environment. A light meter (Dr. Meter LX1010B Digital Illuminance, London, England) is used to measure the illumination change. The illumination is 34 lux and the relative humidity is 49% at the displacement measurement location under the ideal conditions;
Case 2: The truck moves on the bridge while the illumination of the laboratory is changed several times by switching a manual controller. Normally, 9 light panels in the lab are on and the illumination at the measurement location is 34 lux. By turning off the 3 light panels, which are close to the measurement target, the illumination drops to 18 lux. As shown in Figure 6, the left image is lighter, which was taken when the illumination was 34 lux, while the right image is darker which was taken when the illumination was 18 lux;
Case 3: A humidifier (Honeywell HUL520B Mistmate Cool Mist Humidifier, Seattle, WA, USA) is placed between the camera and measuring targets (Figure 7). The humidifier produces a mist at the maximum status to simulate natural fog which decreases the visibility in the camera’s field of view. Normally, the temperature is 24 °C and the relative humidity is 49%. While in the center of the mist, the temperature is 20.3 °C and the relative humidity is 95%.

### 3.2. Results Analysis and Comparative Study of Case 1 (Ideal Case)

**Figure 8:**
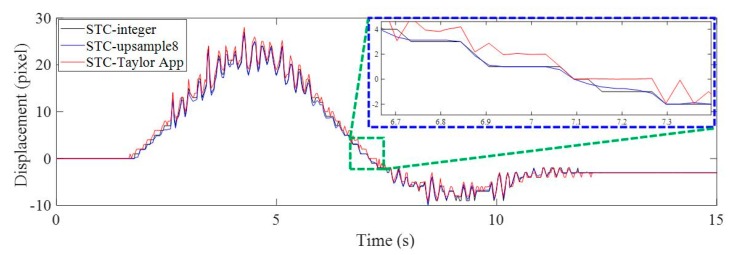
Vertical displacement time histories of P1 in pixel units using non-subpixel, image upsampling, and Taylor approximation techniques.

The objectives for Case 1 were: to evaluate the performance of the subpixel estimation method presented in this paper and verify the feasibility and performance of the proposed method (i.e., STC tracking plus Taylor approximation, STC-Taylor App) by comparing the conventional LP data with the current vision-based displacement methods, e.g., Lucas–Kanade optical flow with SURF features (LK-SURF), key point matching with Fast Library for Approximate Nearest Neighbors and SURF features (FLANN-SURF), and digital image correlation (DIC). Figure 8 illustrates the vertical displacement time histories of P1 in pixel units induced by the vehicle loading when the toy truck traveled along the two-span bridge.

**Figure 9:**
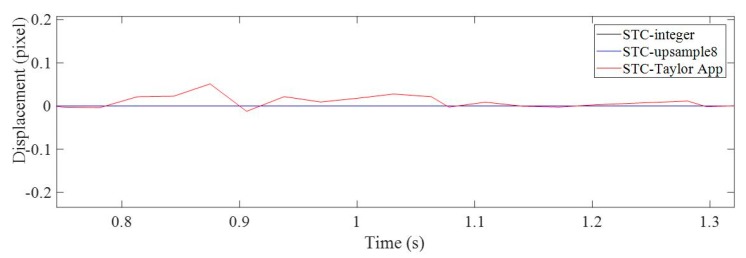
A zoomed in section at the beginning of the vertical displacement time histories of P1.

The vision-based methods used here included STC tracking with non-subpixel (STC-integer), STC tracking with image upsampling (STC-upsample8), and STC tracking with Taylor approximation (STC-Taylor App). In Figure 8, by zooming in on the green, dashed line box area of the displacement time histories, it is clear that the results of the STC-integer approach were rounded to integer values, i.e., (4, 3, 3, 3, 2, 1, 1, 1, 1, 1, 1, 0, 0, (−1), (−1) pixels, …). The image upsampling technique means that each image recorded during the experiment was upsampled 8 times in the horizontal and vertical directions using bicubic interpolation. Then, the minimum resolution was 1/8 = 0.125 pixel. The result of using image upsampling was a much smoother curve and more subpixel-level displacement records. However, it still cannot provide more details about small motions [2], especially at the very beginning and at the end. When there are no apparent loads on the structure, there is still very small structural motion induced by random environmental loads, such as wind, nearby machine operations, ambient ground vibration, etc. As illustrated in Figure 9, during the first several seconds before the toy truck moves, the displacements measured by both STC-integer and STC-upsample8 were exactly zero, which might not be true. Even though the structure was not loaded, it can still vibrate under random environmental loading. The STC-Taylor App indicated the small motions of the structure caused by random environmental loadings. This indicates that the proposed method which combines STC tracking and Taylor approximation has a higher sensitivity, resolution, and accuracy.

**Figure 10:**
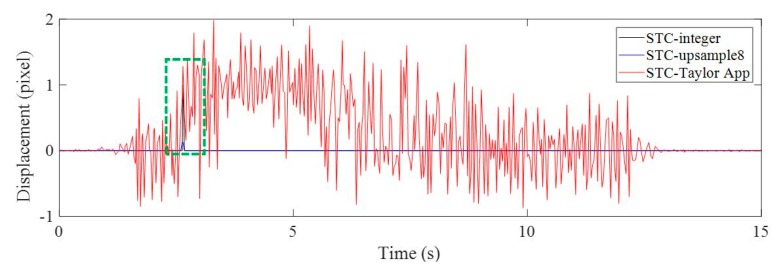
Horizontal displacement time histories of P1 in pixel units using non-subpixel, image upsampling, and Taylor approximation techniques.

**Figure 11:**
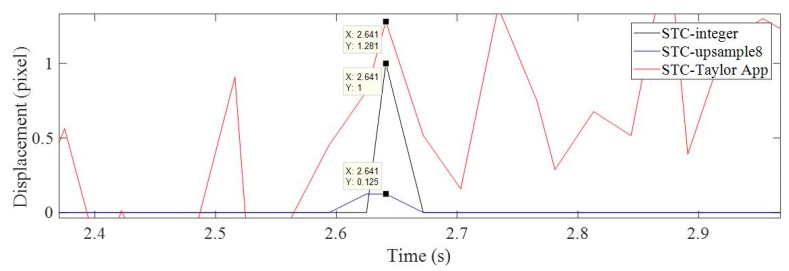
A zoomed in section of the horizontal displacement time histories of P1 in pixel units.

Figure 10 verifies the previous findings. In this figure, the horizontal time displacement histories showed the bridge motion in the longitudinal direction induced by the moving truck impact. The motion was very small, around 1 pixel. The result from the proposed method (STC-Taylor App) provided very detailed information about the vehicle impact, while the results of the STC-integer and STC-upsample8 were almost zero except for one or two points, which means the bridge was stationary in the longitudinal direction. Figure 11 depicts a zoomed in area of the green, dashed line box in Figure 10.

In the zoomed in Figure 11, the displacements of the non-zero points measured by STC-integer, STC-upsample8, and STC-Taylor App were 1, 0.125, and 1.281 pixels. For STC-integer and STC-upsample8, 1 and 0.125 were their minimum measurement resolutions, and statistically, these points were outliers which should be removed from the displacement time histories. In addition, the image processing speed of the proposed method was much faster than using image upsampling.

Table 1 shows the elapsed processing time of one image using three different STC-based methods. The program environment was MATLAB running on a computer with the CPU of i7, 8 processors, and 16 G RAM. The original image had a resolution of 1280 × 960. It took 0.0481 s to process one image to obtain the displacement at the integer pixel level (STC-integer). However, when conducting subpixel level estimation using image upsampling, it took 2.4895 s, which was about (2.4895 − 0.0481)/0.0481 = 50.76 times that of the STC-integer. It took only 0.0495 s to conduct this and provided even better subpixel results when using STC-Taylor App. The proposed method was about 50 times faster than using image upsampling techniques.

Overall, it is suggested that the proposed method using STC tracking and Taylor approximation can provide displacement measurements at the subpixel level with high sensitivity, resolution, accuracy, and faster speed.

**Figure 12:**
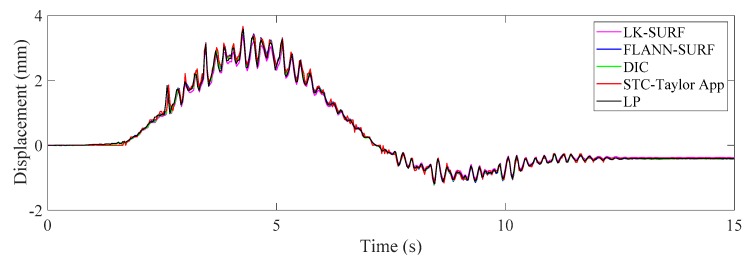
Case 1 (ideal condition): displacement time histories of P1 obtained from different methods.

The next step is to convert the displacement in pixel units to physical units (e.g., millimeter) and verify the feasibility and performance of the displacement measurement by comparing the vision-based methods with the conventional displacement sensor. As illustrated in Figure 12, four vision-based displacement measurement methods (i.e., LK-SURF, FLANN-SURF, DIC, and STC-Taylor App) and one conventional displacement sensor (i.e., LP) were used to obtain the displacement time histories of P1 when the toy truck passed over the bridge. At the very beginning, the toy truck stood at the left end of the first span, then moved to the right and approached the measurement point P1. In the meantime, the displacement of P1 (the downward direction was positive) gradually increased to a maximum when the truck was located at P1. Then the toy truck began to drive off P1 and kept heading to the right, while the displacement of P1 gradually decreased. When the toy truck moved to the right span, the displacement began to be negative (i.e., upward displacement) due to the loading on the other span of the two-span bridge. As it approached the right end of the right span, the absolute value of the displacement at P1 first increased and achieved a maximum and then decreased. When the toy truck arrived at the right end of the bridge, the displacement of P1 became stable but did not go back to zero. This was because the rear axle still rested on the bridge.

By comparing the displacement time histories, it is easy to see that the result obtained from the proposed method (i.e., STC-Taylor App) was quite consistent with those obtained from the LP and other three vision-based methods.

**Figure 13:**
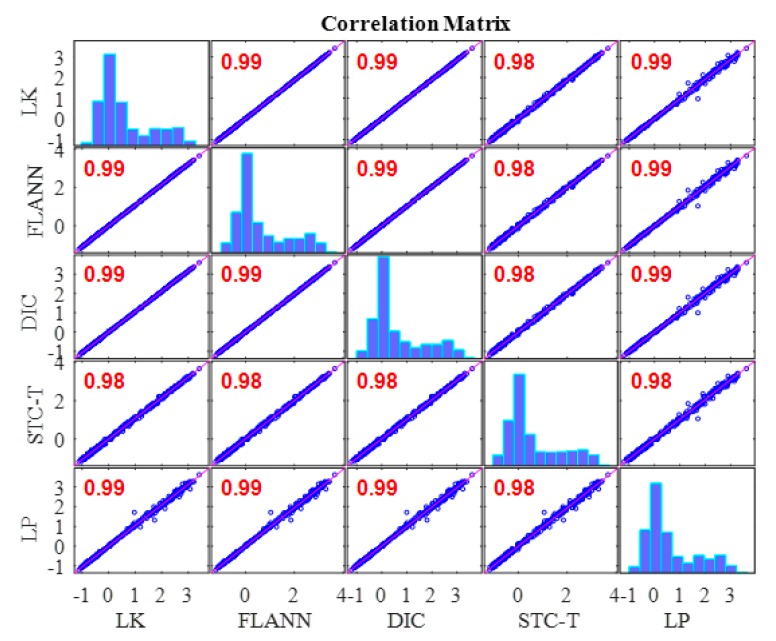
Correlation matrix of time displacement histories of Case 1 (ideal condition).

Figure 13 illustrates the correlation matrix of these time displacement histories for Case 1. The figures on the diagonal of the correlation matrix are the histograms of the displacement time histories, whereas the others are data plots and linear fits among the displacement time histories from the two methods. The correlation matrix is symmetric, and the last row and the last column give the correlation coefficients between the displacement data obtained from the vision-based methods and the conventional displacement sensor, i.e., LP. The correlation coefficients of the LK-SURF, FLANN-SURF, and DIC with the ground truth (i.e., LP) were all 0.99, while the correlation coefficient between the proposed method (i.e., STC-Taylor App) and LP was 0.98, which is also quite good. The performance of the vision-based displacement measurement methods can also be obtained from the similarity of the histograms of each method comparing with the one of LP. Here, from the diagonal element of the correlation matrix, it is indicated that the histograms of these time displacement histories were highly consistent with each other.

**Figure 14:**
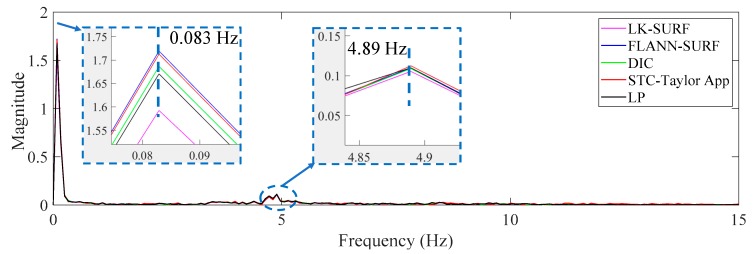
Displacement comparison in the frequency domain of Case 1.

Figure 14 shows the displacement comparison in the frequency domain of Case 1. The first set of peaks from the different methods which gave a frequency value of 0.083 Hz was not the structural mode. The frequency was related to the vehicle loading procedure on the two-span bridge, which produced a sinusoidal shape displacement time history. It seems to be a response cycle and causes the pseudo vibration mode. The frequency value here was related to the speed of the loading. Since the speed of the truck was manually controlled and in each case the speed was different, the frequencies of the first set of peaks were different. This can be observed from Figures 17 and 22 in the following sections. The second set of peaks around 5 Hz (actually 4.87 Hz) was the structural operational vibration frequency corresponding to the higher modes in the displacement time histories in Figure 12. This operational frequency was related to the roughness of the deck and speed of the loading truck. From this figure it can be seen that the structural operational vibration frequencies obtained from different methods were quite consistent.

In this case, under ideal experimental conditions and no adverse factors added to the experiment, the robustness and advantages of the proposed method (STC-Taylor App) did not reveal itself. In the next two cases, the robustness and advantages of the proposed method will be verified.

### 3.3. Results Analysis and Comparative Study of Case 2 (Illumination Change)

**Figure 15:**
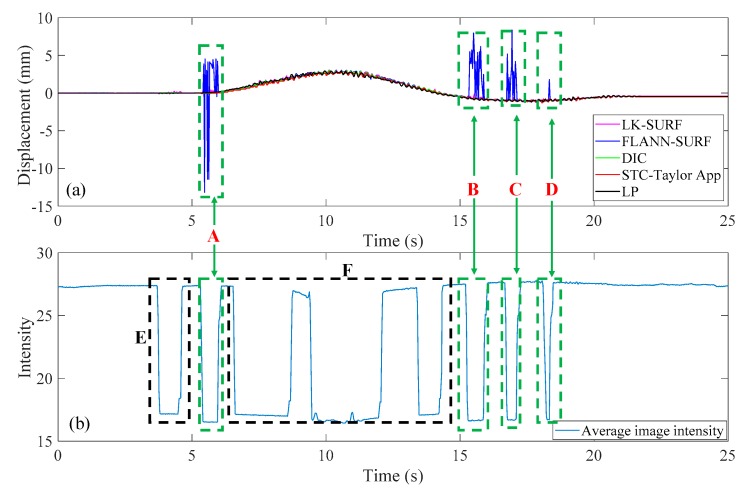
Case 2 (illumination change): (**a**) displacement time histories of P1 obtained from different methods and (**b**) average image intensity time history.

This case was designated to verify the robustness of the proposed vision-based displacement method under the adverse environmental condition: Illumination change. For vision-based methods, illumination is a serious problem when conducting field applications since the image quality is easily affected by illumination change. Consequently, the visual tracking performance and the displacement measurement accuracy are affected by the poor quality in the formation of images. In this experiment, the environmental illumination was determined by the fluorescent light in the lab. By turning the light switches in the laboratory on and off, the image quality changed, as shown in Figure 6. The time histories of P1 obtained from different vision-based methods and LP under environmental illumination change are illustrated in Figure 15a.

**Figure 16:**
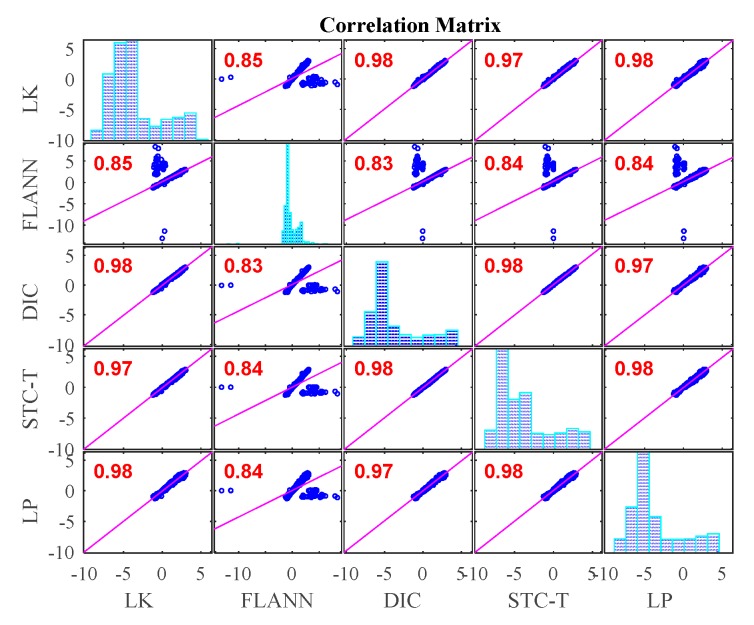
Correlation matrix of time displacement histories of Case 2 (illumination change).

The spikes in Figure 15a show that the vision-based method FLANN-SURF was apparently influenced by the illumination change, which means FLANN-SURF cannot handle this kind of situation compared to other vision-based methods. Figure 15b shows the average image light intensity time history and it is easy to see the illumination change (36 lux to 18 lux) from the tooth-shaped signal. The green, dashed boxes A, B, C, and D indicate the corresponding illumination changes when FLANN-SURF gives spikes in its displacement measurement result. It is suspected that for the illumination changes as shown in boxes E and F, FLANN-SURF shows good consistency with other displacement measurement methods, while the possible reasons are unknown. As shown in Figure 16, the correlation coefficient of the time histories between that obtained from FLANN-SURF and the ground truth, LP, dropped to 0.84, while LK-SURF’s and DIC’s dropped from 0.99 to 0.98 and from 0.99 to 0.97 when compared with the correlation matrix obtained in Case 1, shown in Figure 13. However, the correlation coefficient of the time histories between that obtained from the proposed method, STC-Taylor App, and the ground truth, LP, was still 0.98 compared to that of Case 1.

**Figure 17:**
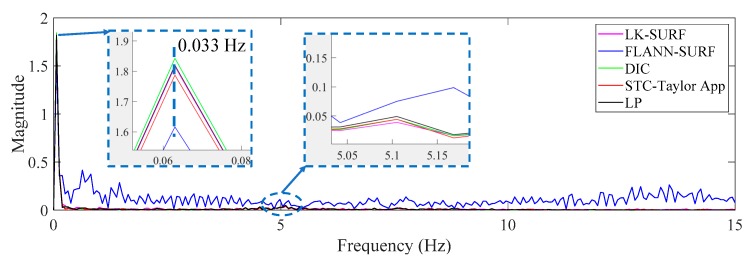
Displacement comparison in the frequency domain of Case 2 (illumination change).

From Figure 15 and Figure 16, it is indicated that the illumination change did have a significant negative effect on FLANN-SURF and might also influence LK-SURF and DIC slightly. On the other hand, the proposed method, STC-Taylor App, showed great robustness and was almost not influenced by the illumination change. This observation can also be found from the frequency domain, as shown in Figure 17. The method FLANN-SURF showed lots of pseudo vibration frequencies which were apparently not the structural vibration modes. The first frequency, 0.033 Hz, indicated the loading cycle, which was similar to Case 1. The structural vibration mode around 5 Hz can barely be seen here, which may be caused by the lower truck speed compared to Case 1. The FLANN-SURF method showed several big pseudo peaks around 5 Hz, while the other vision-based methods kept consistent with LP. The STC-Taylor App could be a good option for long-term vision-based displacement measurement since illumination change is a common problem in field applications.

### 3.4. Results Analysis and Comparative Study of Case 3 (Fog Interference)

**Figure 18:**
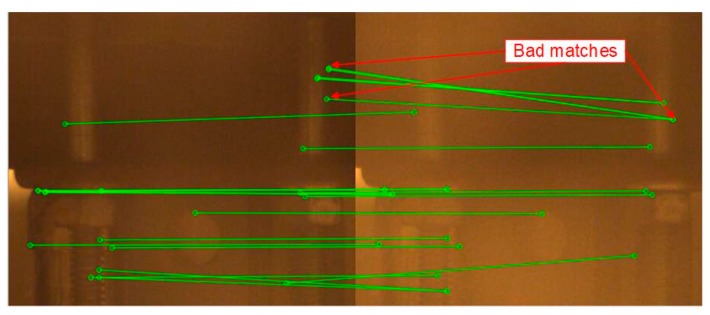
Poor matches when using feature-based methods (fog interference).

This case was designated to verify the robustness of the proposed vision-based displacement method under the adverse environmental condition: fog. In this experiment, the fog was simulated by the mist produced by the humidifier, as shown in Figure 7. The fog not only affected the image quality but also contaminated the image features, which are the basic foundation of target recognition for visual tracking. In addition, the fog was not still but had a random motion. The DIC method may have performed undesirably because it highly relied on the image intensity to conduct pattern matching and the intensity would always change under this situation. Due to the random motion of the fog, a false optical flow would be added to the real target motion which causes the optical flow method (e.g., LK method) to fail. Even though feature points, e.g., Shi-Tomasi corners, SURF, SIFT, FREAK, etc., are very robust and distinctive, their use with feature-based methods (e.g., LK-SURF and FLANN-SURF) still can result in a bad performance due to the presence of bad matches. The mist could block features and induce more bad matches as shown in Figure 18. It causes displacement measurement to have errors, especially when there are not enough feature points to describe the tracking targets.

**Figure 19:**
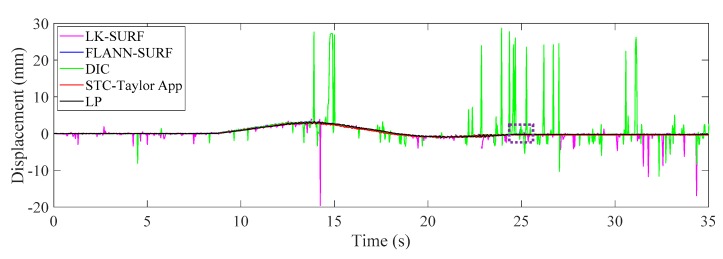
Case 3 (fog interference): displacement time histories of P1 from different methods.

**Figure 20:**
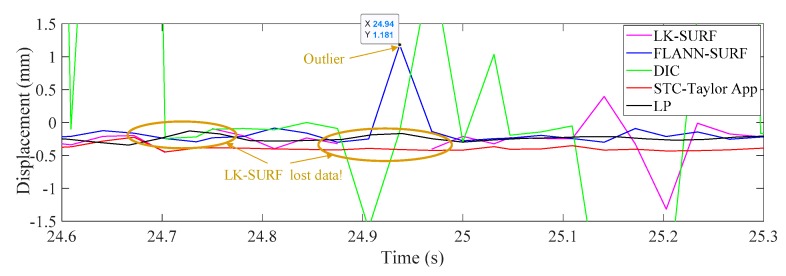
Zoomed in section of the horizontal displacement time histories of P1.

Figure 19 illustrates the time histories of P1 obtained from different vision-based methods and LP under fog interference. When the fog was imposed to the measurement environment, the displacement results obtained from LK-SURF and DIC provided very poor performance resulting in many spikes appearing in the displacement time histories. Only the results from the proposed method (STC-Taylor App) and FLANN-SURF showed satisfactory performance. Figure 20 zooms in on the purple, dashed line box area of Figure 19 and provides more details. In this figure, except the spikes, some data were also lost from the displacement time history, because the visual tracker based on LK-SURF lost the targets due to the fog interference. In general view, even though FLANN-SURF gives a good result, it still has outliers. Figure 20 shows an example of an outlier when using FLANN-SURF. The outlier caused more than a one-millimeter error compared with the ground truth and the result from STC-Taylor App. Statistically, it can be removed.

**Figure 21:**
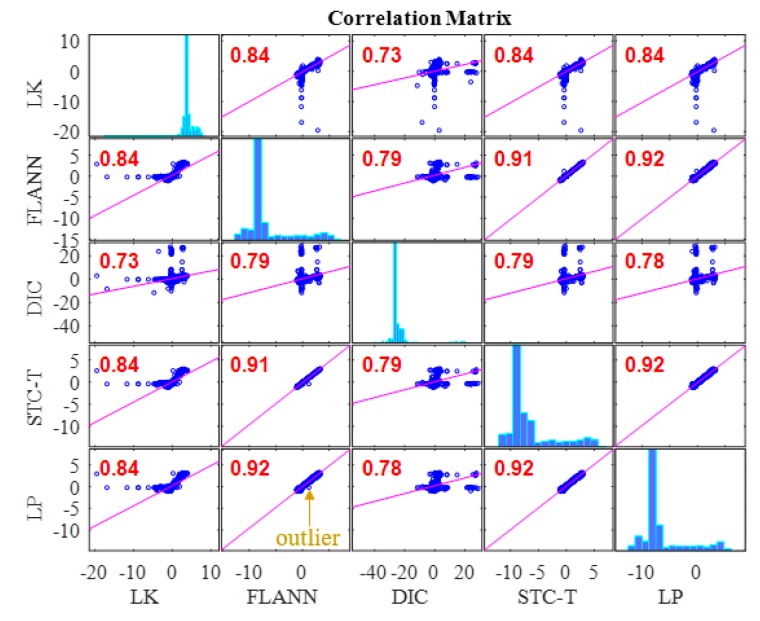
Correlation matrix of time displacement histories of Case 3 (fog interference).

Figure 21 shows the correlation matrix between the vision-based methods and the conventional displacement method, i.e., LP. The correlation coefficient between LK-SURF and LP dropped from 0.99 (in Case 1) to 0.84, which means the measurement error of LK-SURF increased. It was even worse for DIC, for which the correlation coefficient dropped from 0.99 (in Case 1) to 0.78. The linear fit plots between LK-SURF and LP and that between DIC and LP are hard to be interpreted as correlation. The correlation coefficient between the proposed method, STC-Taylor App, and LP also dropped from 0.98 (in Case 1) to 0.92. Considering the initial status, it is a little bit better than that of FLANN-SURF, since the correlation coefficient between the proposed method, STC-Taylor App, and LP also dropped from 0.99 (in Case 1) to 0.92. The outlier in the displacement time history obtained using FLANN-SURF also showed correlation in matrix plot. The fog indeed had undesirable effects on all of these vision-based methods at different levels. These bad effects might not be easy to find or be quantified in the time histories, but they apparently reveal themselves in correlation matrix.

**Figure 22:**
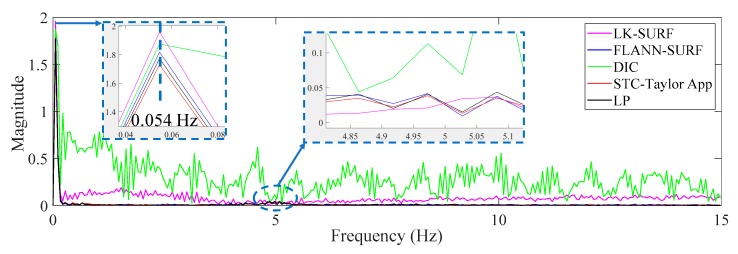
Displacement comparison in frequency domain of Case 3 (fog interference).

Figure 22 gives the comparison in the frequency domain of Case 3. The poor performance of LK-SURF and DIC caused by fog can also easily be seen from the pseudo peaks (purple and green curves) in the frequency domain. Similar to Case 1 and Case 2, the first set of peaks indicate the loading procedure and that there should be a structural vibration mode frequency peak around 5 Hz; however, it can barely be seen. Except for LK-SURF and DIC, the proposed method (STC-Taylor APP) and FLANN-SURF seemed to match very well with LP, which is consistent with the observations from Figure 19 and Figure 21.

Compared to the other three vision-based methods, the proposed method provided the best performance. The proposed vision-based displacement measurement method, i.e., STC-Taylor App, showed great robustness under fog interference. The STC-Taylor App could be a good option for long-term vision-based bridge displacement measurement since fog is a common weather problem in field application, especially when the bridge crosses a river and during foggy seasons. Considering the results analysis of Case 2 and Case 3, the proposed method shows the best performance under the two adverse environmental factors.

## 4. Conclusions

In this study, a robust non-contact displacement measurement method using spatio-temporal context learning and Taylor approximation was proposed. This study aimed to resolve the adverse effects induced by te environmental factors such as illumination change and fog interference when using vision-based methods to conduct displacement measurements without adding manual markers or artificial light source for long-term bridge monitoring. The first method that was proposed, namely, spatio-temporal context learning, leveraged the advantage of images with high-resolution spatial and temporal aspects, which can be used for long-term bridge monitoring. Then, as an extension, the Taylor approximation technique was implemented into the proposed method to improve the accuracy of the displacement at the subpixel level without sacrificing the processing speed. The performance of the proposed subpixel estimation method was compared with general image upsampling techniques and the results showed that the proposed subpixel estimation method was faster than the general image upsampling techniques by about 50 times. Also, the precision of the proposed method was much better than the general image upsampling technique. To validate the feasibility, stability, and robustness of the proposed method, a series of experiments on a two-span three-lane bridge in laboratory under the adverse environmental factors such as illumination change and fog interference were conducted. The illumination change was achieved by turning on and off the light switches in the room and the fog interference was simulated with a humidifier which could produce mist. The results from the proposed method showed that:

1. In Case 1, there were no adverse environmental factors and the measurement condition was desirable for vision-based systems. The correlation coefficients of the LK-SURF, FLANN-SURF, and DIC with the ground truth, i.e., LP, were all 0.99, while the correlation coefficient between the proposed method, STC-Taylor App, and LP was 0.98, which was also quite good. It indicated that that at least in a desirable measurement environment, the proposed method is a strong competitor of the current methods.
2. In Case 2, with the illumination change, the correlation coefficient of the time histories between that obtained from FLANN-SURF and the ground truth, LP, dropped to 0.84, while LK-SURF’s and DIC’s only dropped from 0.99 to 0.98 and from 0.99 to 0.97, respectively, compared with the correlation matrix obtained from Case 1. However, the correlation coefficient of the time histories between that obtained from the proposed method, STC-Taylor App, and the ground truth, LP, was still 0.98 comparing to that of Case 1.
3. In Case 3, with the fog interference, the correlation coefficient between LK-SURF and LP dropped from 0.99 to 0.84, while DIC’s drops from 0.99 to 0.78, which was the worst performance. The FLANN-SURF’s dropped from 0.99 to 0.92 and the proposed method, STC-Taylor App, dropped from 0.98 to 0.92.

Combining the results analysis of the experiments, the proposed method showed the best performance under the two adverse environmental factors, and it provided an accuracy at the subpixel level without sacrificing the processing speed. By considering the spatial and temporal context learning processes, the proposed method in this paper successfully mitigated the effects induced by illumination change and fog interference. The poor performances of FLANN-SURF in Case 2 and of LK-SURF and DIC in case 3 resulted in spikes in the displacement measurements, which can be removed by using low-pass filtering. However, this would limit the usability of these methods at higher frequencies. The proposed method seems to provide accurate displacements without the need of filtering the results.

Although, the benefits of the proposed method to address other real-world challenges is not explored in this paper, the proposed method may be applied to solve other adverse influencing factors, such as motion blur, rain, object occlusion, out of plane movement, orientation of the camera relative to the bridge and camera motion, etc., by taking advantage of the high spatio-temporal resolution. The computer vision-based approach along with the proposed method can be a good alternative and a complementary approach to conventional structural health monitoring practices. In the future, more studies will be carried out on real bridges to validate the feasibility of the proposed method and also to investigate other relevant challenges for long-term bridge monitoring using computer vision. Besides, in this study, only one camera was used, and the proposed method was verified by tracking the motion of the bridge deck on a two-dimensional (2D) plane, which is a limitation. In future studies, the feasibility of 3D motion tracking using the proposed method will be investigated and will be tested on other applications such as long span bridge monitoring and cable vibration monitoring. Also, the effects of illumination inhomogeneity and non-linear illumination changes to the measurement performance of different vision-based methods will be explored.

## Figures and Tables

**Table 1:** Time consumption of processing one image using different STC-based methods.

Methods	STC-Integer	STC-Upsample8	STC-Taylor App
Time(s)	0.0481	2.4895	0.0495
